# Effect of prenatal micronutrient-fortified balanced energy-protein supplementation on maternal and newborn body composition: A sub-study from the MISAME-III randomized controlled efficacy trial in rural Burkina Faso

**DOI:** 10.1371/journal.pmed.1004242

**Published:** 2023-07-24

**Authors:** Alemayehu Argaw, Laeticia Celine Toe, Giles Hanley-Cook, Trenton Dailey-Chwalibóg, Brenda de Kok, Lionel Ouédraogo, Anderson Compaoré, Moctar Ouédraogo, Amadi Sawadogo, Rasmané Ganaba, Katrien Vanslambrouck, Patrick Kolsteren, Carl Lachat, Lieven Huybregts

**Affiliations:** 1 Department of Food Technology, Safety and Health, Faculty of Bioscience Engineering, Ghent University, Ghent, Belgium; 2 Unité Nutrition et Maladies Métaboliques, Institut de Recherche en Sciences de la Santé (IRSS), Bobo-Dioulasso, Burkina Faso; 3 AFRICSanté, Bobo-Dioulasso, Burkina Faso; 4 Poverty, Health, and Nutrition Division, International Food Policy Research Institute (IFPRI), Washington, DC, United States of America

## Abstract

**Background:**

Micronutrient-fortified balanced energy-protein (BEP) supplements are promising interventions to prevent intrauterine growth retardation in low- and middle-income countries. On the other hand, one concern with blanket prenatal supplementation programs using energy-dense supplements is that they could lead to more maternal and/or infant overweight. However, evidence is lacking on the potential effect of BEP on maternal and offspring body composition. This study evaluates the effects of micronutrient-fortified BEP supplementation during pregnancy on body composition of mothers and their newborns in rural Burkina Faso.

**Methods and findings:**

The MISAME-III study is an open label individually randomized controlled trial where pregnant women (*n* = 1,897) of gestational age <21 weeks received either a combination of micronutrient-fortified BEP and iron-folic acid (IFA) tablets (i.e., intervention) or IFA alone (i.e., control). The prenatal phase of the MISAME-III study was conducted between the first enrollment in October 2019 and the last delivery in August 2021. In a sub-study nested under the MISAME-III trial, we evaluated anthropometry and body composition in newborns who were born starting from 17 November 2020 (*n*: control = 368 and intervention = 352) and their mothers (*n*: control = 185 and intervention = 186). Primary study outcomes were newborn and maternal fat-free mass (FFMI) and fat-mass (FMI) indices. We used the deuterium dilution method to determine FFMI and FMI and %FFM and %FM of total body weight within 1 month postpartum. Our main analysis followed a modified intention-to-treat approach by analyzing all subjects with body composition data available. Univariable and multivariable linear regression models were fitted to compare the intervention and control arms, with adjusted models included baseline maternal age, height, arm fat index, hemoglobin concentration and primiparity, household size, wealth and food security indices, and newborn age (days). At study enrollment, the mean ± SD maternal age was 24.8 ± 6.13 years and body mass index (BMI) was 22.1 ± 3.02 kg/m^2^ with 7.05% of the mothers were underweight and 11.5% were overweight. Prenatal micronutrient-fortified BEP supplementation resulted in a significantly higher FFMI in mothers (MD (mean difference): 0.45; 95% CI (confidence interval): 0.05, 0.84; *P* = 0.026) and newborns (MD: 0.28; 95% CI: 0.06, 0.50; *P* = 0.012), whereas no statistically significant effects were found on FMI. The effect of micronutrient-fortified BEP on maternal FFMI was greater among mothers from food secure households and among those with a better nutritional status (BMI ≥21.0 kg/m^2^ or mid-upper arm circumference (MUAC) ≥23 cm). Key limitations of the study are the relatively high degree of missing data (approximately 18%), the lack of baseline maternal body composition values, and the lack of follow-up body composition measurements to evaluate any long-term effects.

**Conclusions:**

Micronutrient-fortified BEP supplementation during pregnancy can increase maternal and newborn FFMI, without significant effects on FMI.

**Trial registration:**

ClinicalTrials.gov with identifier NCT03533712.

## Abstract 2

In a sub-study nested under the MISAME-III trial, an individually randomised controlled efficacy trial in rural Burkina Faso, Carl Lachat and colleagues investigate the effect of prenatal micronutrient-fortified balanced energy-protein supplementation on body composition of mothers and their newborns.

## Author summary

**Why was this study done?:**

- Prenatal multimicronutrient-fortified balanced energy-protein (BEP) supplementation is a promising nutritional intervention to alleviate the high burden of intrauterine growth retardation, including low birth weight and small-for-gestational age, in low- and middle-income countries.
- On the other hand, one concern with blanket prenatal supplementation programs using energy-dense supplements is that they could lead to more infant and/or maternal overweight, as observed with consumption of ultra-processed foods in high-income countries.
- However, only few studies evaluated the impact of prenatal BEP supplementation on the body composition of mothers and their newborns using a robust RCT design and a direct body composition assessment.

**What did the researchers do and find?:**

- In a sub-study nested under the MISAME-III trial, an individually randomized controlled efficacy trial in rural Burkina Faso, we evaluated the effects of prenatal micronutrient-fortified BEP supplementation on body composition of mothers and their newborns.
- Our trial indicates that micronutrient-fortified BEP supplementation during pregnancy can improve maternal and newborn fat-free mass index (FFMI) without altering maternal fat-mass index (FMI).

**What do these findings mean?:**

- Our findings that prenatal BEP improved FFMI without increases in FMI address the possible concern that prenatal BEP could lead to excess adiposity and associated long-term health risks.
- Future research is required to investigate the long-term effects of prenatal BEP on body composition of older infants and children.

## Introduction

Low birth weight (LBW; i.e., born <2,500 g) is a marker of suboptimal fetal growth and development caused by preterm delivery (i.e., gestational duration <37 weeks) and/or fetal growth restriction, which results in a small-for-gestational age (SGA) newborn (i.e., birth weight below the tenth percentile of a reference group of newborns with the same gestational age). LBW is associated with a significantly higher neonatal mortality risk that varies between a 2-fold risk increment for SGA neonates, a 7-fold risk for preterm newborns, and up to a 15-fold risk increase when children are born both preterm and SGA [1]. Furthermore, LBW is associated with subsequent poor infant and child growth, morbidity, suboptimal early development, and adult chronic disease [2–4]. In 2015, 14.6% of newborns suffered from LBW as compared to 17.5% in 2000 with more than 90% of all LBW occurring in low- and middle-income countries [5]. Without additional efforts, it is unlikely that the World Health Assembly nutrition target to reduce LBW by 30% between 2012 and 2025 will be achieved.

Prenatal nutritional supplementation is one key strategy to prevent LBW. Systematic review and meta-analysis of relevant studies showed that prenatal multimicronutrient (MMN) fortified balanced energy-protein (BEP) supplementation reduces the risk of SGA (relative risk (RR): 0.79; 95% confidence interval (CI): 0.69, 0.90) [6], while prenatal food distribution was associated with an apparent reduction in LBW (RR: 0.92; 95% CI: 0.84, 1.0) [7]. One particular variety of MMN-fortified BEP supplements, known as lipid-based nutritional supplements (LNS), consists of promising food matrix to deliver the necessary macro- and micronutrients during gestation. LNS are typically in the form of peanut-based spreads that have low water activity, which prevents microbial proliferation, and are conveniently ready-to-consume. Two individually randomized controlled efficacy trials in Burkina Faso (i.e., MISAME-II and MISAME-III) assessed the effect of prenatal LNS on birth outcomes. Results of these trials suggest small to modest effects on birth length (MD (mean difference): 4.6 mm; 95% CI: 1.8, 7.3) in MISAME-II [8], and on birth weight (MD: 49.7 g; 95% CI: 10.8, 88.7) and birth length (MD: 2.0 mm; 95% CI: 0.1, 3.9) in MISAME-III [9]. In addition, the MISAME-III trial only found a marginal effect of BEP on gestational weight gain (0.28 kg; 95% CI: −0.05, 0.58 kg) [10].

One concern with blanket prenatal supplementation programs using energy-dense supplements, such as LNS, is that it could lead to more infant and/or maternal overweight, as observed with consumption of ultra-processed foods in high-income countries [11]. A clinical sub-study nested in the MISAME-II trial comparing prenatal LNS to MMN supplements found that LNS led to a higher cord blood leptin concentration [12]. Leptin in cord blood originates primarily from the fetal compartment and is a marker of neonatal fat mass [13,14]. The observed effect on cord leptin concentration was exacerbated in women with a higher body mass index (BMI) or mid-upper arm circumference (MUAC) at early gestation and was associated with a positive effect on birth weight. To further investigate this exploratory finding, we designed a sub-study nested in the main MISAME-III trial with as primary objectives (i) to assess if prenatal LNS altered neonatal body composition; and (ii) to examine if maternal nutritional status at early gestation modifies any effect of prenatal LNS on neonatal body composition. In addition, to address possible concerns related to prenatal BEP and maternal overweight or adiposity, we also assessed if prenatal LNS led to an effect on postpartum maternal body composition.

## Methods

### Ethics statement

The MISAME-III study protocol was approved by the ethics committee of Centre Muraz in Burkina Faso (N°2018–22/MS/SG/CM/CEI) and Ghent University Hospital in Belgium (B670201734334). Participant mothers provided their written consent for participation after an information session detailing the study, voluntary participation, and withdrawal. The trial is registered at ClinicalTrials.gov with registration number NCT03533712.

### Study design and intervention

The MISAME-III study is an individually randomized 2 × 2 factorial trial evaluating the efficacy of a combination of micronutrient-fortified BEP and iron-folic acid (IFA) supplementation, as compared to IFA supplementation alone, during pregnancy and during lactation on various maternal, newborn and infant outcomes. In this sub-study nested in the main MISAME-III trial, we evaluate the effect of prenatal micronutrient-fortified BEP supplementation on maternal and newborn body composition.

The MISAME-III parent study has been described in detail previously [9,10,15]. In brief, the study was implemented in 6 rural health center catchment areas in the district of Houndé in the Hauts-Bassins region of Burkina Faso. The study area is characterized by a Sudano-Sahelian climate with a dry season running between September/October and April, and agricultural activities being the main livelihood of the community. The habitual diet during pregnancy is nondiverse [16], predominantly based on maize with a complement of leafy vegetables [17]. The mean daily energy intake from the base diet (i.e., excluding supplements) during pregnancy was estimated to be approximately 1,940 kcal in a subsample of MISAME-III participants at the end of the preharvest season, and dietary micronutrient intakes were inadequate to cover the estimated average requirements (EARs) [18]. During the previous MISAME-II trial, baseline prevalence of maternal underweight (BMI <18.5 kg/m^2^) during pregnancy was 12.9% [8].

Women were randomly assigned to receive either daily BEP and IFA supplementation (intervention) or IFA alone (control). The BEP supplement was a large-quantity LNS in the form of an energy-dense peanut paste fortified with multiple micronutrients [9]. A daily dose of the BEP (72 g) consisting of 36% lipids, 20% protein, and 32% carbohydrates provided an energy top-up of 393 kcal (**S1 Table**). The supplement covered at least the EARs of pregnant women for 11 micronutrients [19]. A daily dose of an IFA tablet (Sidhaant Life Sciences, Delhi, India) contained 65 mg iron [form: FeH_2_O_5_S] and 400 μg folic acid [form: C_19_H_19_N_7_O_6_]. Supplementation was provided under direct observation by locally trained village-based project workers conducting daily home visits of study participants.

### Study participants

The prenatal phase of the main MISAME-III study was conducted between the first enrollment on 30 October 2019 and the last delivery on 7 August 2021. Pregnant women (*n* = 1,897) aged between 15 and 40 years with a gestational age less than 21 completed weeks were enrolled into the study. Women who reported an allergy to peanuts, who planned to leave or deliver outside of the study area, and who had multifetal pregnancy were excluded from the study. Data collection for the body composition sub-study was started on 17 November 2020, enrolling the 880 newborns delivered from that date onwards. From this sample, a random subsample of 390 mothers enrolled in this study. The random selection of mothers was generated by the Survey Solutions tablet-assisted personal interviewing software.

### Stable isotope administration and saliva sample collection

The body composition of mothers and infants was assessed using the stable isotope dilution technique with deuterium oxide (D_2_O) [20]. Mother-child pairs were invited to the health center during the third week postpartum. If the mother was absent at the scheduled visit, project village workers would conduct a home visit to invite the mother to attend 1 week later. In total, 3 saliva samples were collected from each subject using small cotton balls. First, a first baseline saliva sample was collected followed by an administration of the deuterium tracer. A pre-weighed constant dose of 1 g (dose volume of 0.9 mL) and 6 g (dose volume of 5.4 mL) of D_2_O (99.8%, Sigma-Aldrich, St. Louis, Canada) was administered to infants and mothers, respectively. The doses of D_2_O were weighed in a laboratory in Bobo-Dioulasso, to the nearest 0.0001 g, using a precision balance (model SAB 224i, Adams Equipment, Felde, Germany). Then, a second saliva sample was collected 2 h and 30 min after administering the tracer for children and 3 h for mothers. Lastly, a third saliva sample was collected 30 min after the second sample. Each neonatal and maternal saliva sample was approximately 1.5 to 2 mL in volume. For newborns, the cotton balls were wrapped around a straw and saliva was collected by swabbing the inside of the neonate’s cheeks. Mothers were asked to chew on the cotton balls to moisten them. Cotton balls were expressed in 20 mL disposable syringes and the saliva was collected in a 2 mL disposable cryovial. The cryovials were labeled and stored in a zip lock bag in a portable cooler (2 to 6°C). After completion of the sample collection, samples were transported to the project office in Houndé and stored in a dedicated freezer at −18°C. Samples were transported in cool cryoboxes from the project office to the laboratory in Bobo-Dioulasso once every week where they were stored at −20°C until the time of analysis.

In the event that both the mother and her newborn were selected (*n* = 390) for the sub-study, the D_2_O dose for the mother was administered after the collection of the last neonatal saliva sample to avoid any transfer of D_2_O tracer from the mother to the newborn through breastmilk. Breastmilk was expressed using a medical-grade breast pump (Medela, Mississauga, Canada) into sterile baby bottles, labeled and stored in a cool box. Mothers were allowed to bottle-feed their child during the 2.5 h equilibration phase (between the second and first saliva sample). The net weight of the milk consumed was recorded using scales with a precision of 1 g (model KD7000, www.myweigh.com). The field experiment and procedures were pilot tested in 12 mother-infant dyads.

The D_2_O concentrations in saliva samples were measured by Fourier-transformed infrared (FTIR) spectrophotometry using an Agilent 4500 Series device (Agilent, California, United States of America). All samples were measured in duplicate. The mean of the 4 measurements of the post-administration saliva samples was used for analysis. A calibration curve was compiled prior to every measurement session to convert the signal into a D_2_O concentration. The isotopic enrichment of saliva was calculated by subtracting the quantity of D_2_O measured in the baseline sample from that measured in the post-dose samples. The D_2_O dilution volume was calculated by dividing the administered dose of D_2_O by the isotopic enrichment. Total body water (TBW) was calculated by multiplying the D_2_O dilution volume by 1.044 to account for 4.4% proton loss. For newborns who were breastfed between sample collections, we subtracted the weighed quantity of consumed breastmilk multiplied by 87%, which is the estimated water content of breastmilk [21], from the TBW. Fat-free mass (FFM) in kg was calculated from TBW using age- and sex-specific hydration factors for newborns [22] and mothers [23]. Fat mass (FM) was calculated by subtracting the FFM from total body weight.

### Study outcomes and measurement

The primary outcomes of interest of the study are fat-mass index (FMI) and fat-free mass index (FFMI) (i.e., mass divided by squared height) in mothers and newborns. Secondary study outcomes are %FFM and %FM of total body weight in mothers and newborns; maternal BMI; and newborn length-for-age, weight-for-age, and weight-for-length z-scores. A sample size of 440 newborns per study arm (880 in total) was required to detect an effect size (Cohen’s d) of 0.2 on FFMI and 0.4 for an interaction effect between supplementation and maternal low versus high BMI (<21 versus ≥21 kg/m^2^) or MUAC (<23 versus ≥23 cm) with a type I error of 5%, a statistical power of 80%, and assuming 10% sample loss. Furthermore, a sample size of 195 women per study arm (390 in total) was required to detect an effect size (Cohen’s d) of 0.3 on FFMI with a type I error of 5%, a statistical power of 80%, and assuming 10% sample loss.

Body composition assessment of mothers and newborns was conducted within 3 weeks postpartum (mean ± SD days after delivery: 18.2 ± 3.96). Newborn weight was measured to the nearest 10 g using a Seca 384 scale, and length was measured to the nearest 1 mm using a Seca 416 length board. Maternal weight was measured to the nearest 100 g using a Seca 876 scale and height was measured to the nearest 1 mm using a ShorrBoard. All measurements were taken in duplicate and a third measurement was taken when there was a large discrepancy between the duplicate measurements. We used the average of the 2 closest anthropometric measurements for analysis. Anthropometric indices of length-for-age, weight-for-age, and weight-for-length *z*-scores were calculated based on the World Health Organization 2006 Child Growth Standards [24]. Household food security status was evaluated using Household Food Insecurity Access Scale [25], and the UNICEF/WHO water and sanitation tool was employed to assess access to improved drinking water and improved sanitation facilities [26]. Household wealth status was assessed by a wealth index score generated using the principal components analysis based on availability of household assets, facilities, and housing conditions.

### Statistical analysis

Data analyses were conducted using Stata 17.1 (Statacorp, Texas, USA) and a two-sided statistical significance was considered at alpha <0.05. Descriptive statistics are presented as means ± SD for the continuous variables and as frequencies (%) for the categorical variables. Study outcomes were checked for normality of distribution using visual inspection of histograms and Q-Q plots of the outcome values and their residual terms.

Unadjusted and adjusted group differences in body composition indices were estimated by fitting linear regression models. All models included health center and randomization block as covariates to account for the study design [15]. Adjusted regression models additionally included prespecified known prognostic factors of the outcomes and factors that are associated with body composition indices and that differ between study arms by >2.5%. These included maternal age, height, arm fat index, hemoglobin concentration and primiparity at study enrollment, household size, wealth index and food security index, and the number of postpartum days before the body composition measurement. The main analysis followed a modified intention-to-treat approach by analyzing all participants having body composition measurements. Outcome values that were found to be extreme (i.e., standardized values >abs[3 *z*-scores]) were inspected individually and excluded from data analysis if deemed implausible. We also conducted a per-protocol analysis by comparing only participants with a BEP adherence of at least 75% with the control subjects. Following a suggestion of one reviewer of the manuscript, we deviated from our statistical analysis plan replacing the naïve per-protocol analysis by a more robust complier average causal effect (CACE) analysis. The CACE approach has advantage over the per-protocol analysis in maintaining the balance between treatment groups obtained through the randomization process by comparing compliers in the BEP group versus “inferred compliers” (would be compliers) in the control group [27]. For this purpose, using the *gsem* command in Stata [28], we applied a latent class regression modeling approach simultaneously fitting models for compliance and for the intervention effect. Compliance status was predicted by covariates such as household food insecurity, access to improved water and sanitation, number of under-five children, and number of job activities by the women and the household head, while the effects of BEP on the study outcomes were estimated by adjusting for the aforementioned covariates.

Besides evaluating any effect modification of BEP by maternal BMI and MUAC at study enrollment, we explored the presence of effect modification by other covariates. For this purpose, we tested an interaction effect of the intervention and each factor on FFMI and FMI. Effect modification by a subgroup factor was considered in the presence of a statistically significant interaction (*P* < 0.10). We also applied the approach by Katz and colleagues [29] to evaluate whether the treatment effect of prenatal BEP on FFMI and FMI was comparable between mothers and newborns across the maternal BMI distribution. In this approach, differences in maternal and newborn FFMI and FMI between intervention and control groups are estimated as nonlinear smooth functions of the percentiles of maternal BMI distribution at baseline. Lastly, we fit linear regression models to evaluate the relationships between maternal and newborn FFMI, and between FFMI and FMI and birth anthropometry.

## Results

**Fig 1:**
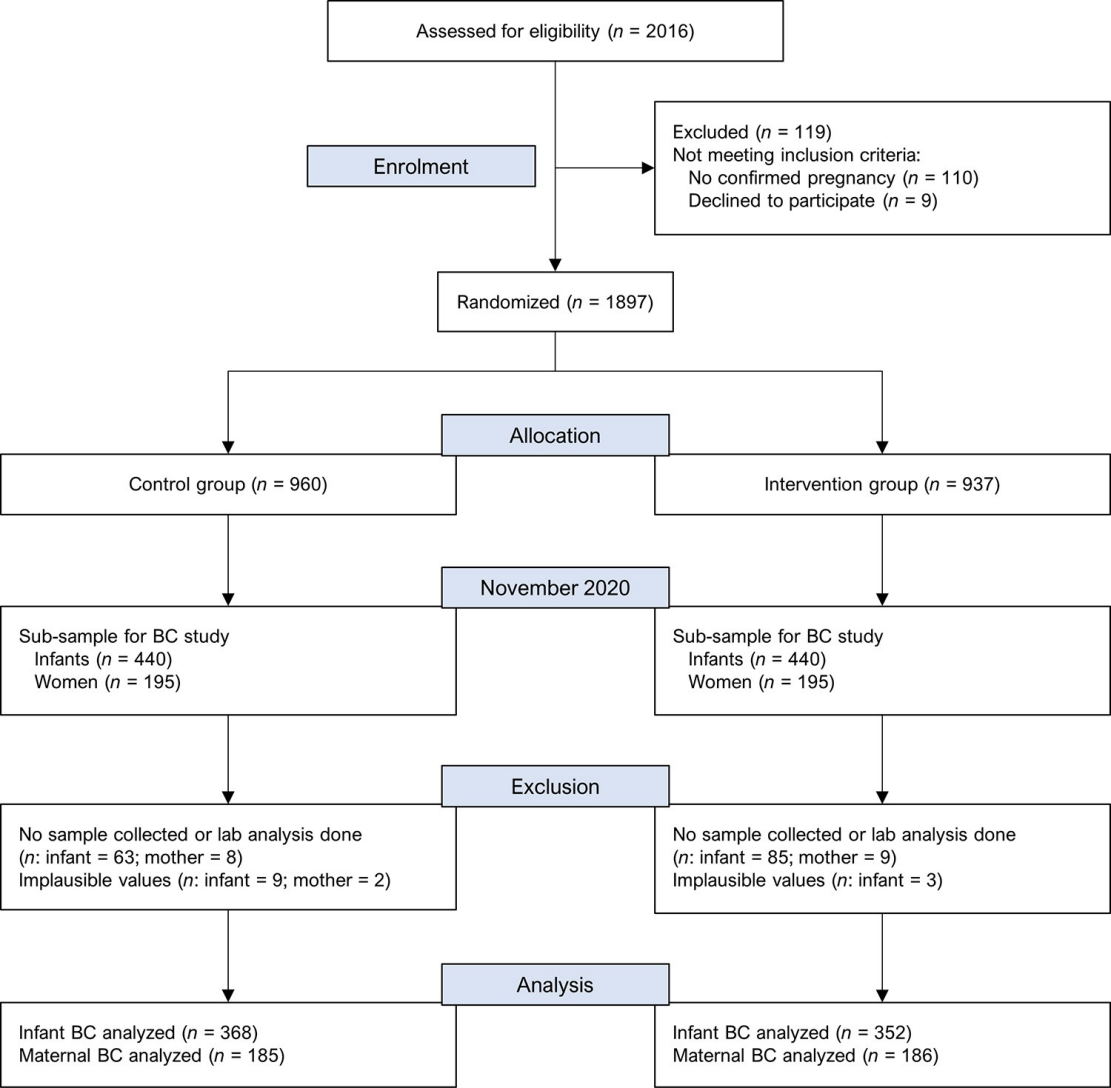
Trial flow diagram of the MISAME-III body composition sub-study. BC, body composition; MISAME, MIcronutriments pour la SAnté de la Mère et de l’Enfant.

From the 880 newborns considered for the body composition sub-study, we analyzed data from 720 (81.8%) newborns (**Fig 1**). One hundred and sixty newborns were not included in the analysis due to a lack of sample collection or lab analyses not being conducted (*n* = 148) or implausible values in anthropometric or body composition outcomes (*n* = 12). We analyzed data from 371 (95.1%) of the 390 mothers selected for the current sub-study, whereas the remaining 19 mothers were not included in our analysis due to a lack of sample collection or lab analyses not being conducted (*n* = 17) or implausible anthropometric or body composition values (*n* = 2). **Table 1** presents the characteristics of participants in the newborn and maternal body composition sub-studies. Study groups were comparable with regard to baseline household and maternal characteristics in both the newborn and maternal body composition study samples. Participant characteristics in the newborn and maternal body composition sub-studies were also similar to those from the main MISAME-III study (**S2 Table**). Mean ± SD maternal age in the newborn subsample was 24.8 ± 6.13 years with 41.7% of participants having had 3 or more pregnancies and 58.6% were from food-insecure households. At enrollment, during the first and early second trimester of pregnancy, mean ± SD maternal BMI, MUAC, and arm fat index were 22.1 ± 3.02 kg/m^2^, 26.2 ± 2.64 cm, and 25.5 ± 7.26, respectively, and 7.05% of the mothers were underweight (BMI <18.5 kg/m^2^) and 11.5% were overweight (BMI ≥25.0 kg/m^2^). We observed a high adherence of mothers to both the BEP (84.2%) and IFA (89.3%) supplements, with compliance to IFA supplement being comparable between control (88.4%) and intervention (90.3%) groups.

**Table 1:** Participant characteristics between study arms in the newborn and maternal body composition study^1^.

Characteristics	Infant sample		Maternal sample	
	Control (*n* = 440)	Intervention (*n* = 440)	Control (*n* = 195)	Intervention (*n* = 195)
**Health center catchment area**				
Boni	110 (25.0)	102 (23.2)	45 (23.1)	49 (25.1)
Dohoun	38 (8.60)	44 (10.0)	21 (10.8)	25 (12.8)
Dougoumato II	88 (20.0)	82 (18.6)	34 (17.4)	32 (16.4)
Karaba	42 (9.50)	47 (10.7)	21 (10.8)	16 (8.20)
Kari	84 (19.1)	93 (21.1)	35 (17.9)	34 (17.4)
Koumbia	78 (17.7)	72 (16.4)	39 (20.0)	39 (20.0)
**Household level**				
Wealth index, 0 to 10 points	4.53 ± 1.72	4.74 ± 1.76	4.51 ± 1.69	4.69 ± 1.74
Household food insecurity^2^	257 (58.4)	260 (59.1)	119 (61.0)	110 (56.4)
Improved primary water source^3^	268 (60.9)	273 (62.0)	106 (54.4)	122 (62.6)
Improved sanitation facility^4^	260 (59.1)	241 (54.8)	109 (55.9)	112 (57.4)
Household size	6.44 ± 4.52	6.47 ± 4.62	6.49 ± 4.59	6.63 ± 4.82
Polygamous households	129 (29.3)	129 (29.3)	62 (31.8)	55 (28.2)
**Maternal**				
Age, years	25.1 ± 6.11	24.9 ± 6.11	25.1 ± 5.99	24.7 ± 5.97
Ethnic group				
Bwaba	261 (59.3)	254 (57.7)	115 (59.0)	104 (53.3)
Mossi	148 (33.6)	149 (33.9)	74 (37.9)	72 (36.9)
Others	31 (7.05)	37 (8.41)	6 (3.08)	19 (9.74)
Religion				
Muslim	178 (40.5)	185 (42.0)	82 (42.1)	92 (47.2)
Animist	105 (23.9)	99 (22.5)	44 (22.6)	36 (18.5)
Protestant	88 (20.0)	86 (19.5)	36 (18.5)	37 (19.0)
Catholic	56 (12.7)	54 (12.3)	30 (15.4)	26 (13.3)
No religion, no animist	13 (3.00)	14 (3.20)	3 (1.50)	4 (2.10)
Primary education and above	189 (45.0)	181 (41.1)	90 (46.2)	89 (45.6)
Gestational age, weeks	11.0 ± 3.87	10.9 ± 3.86	10.9 ± 3.86	10.7 ± 3.84
Trimester of gestation				
First	295 (67.0)	299 (68.0)	136 (69.7)	132 (67.7)
Second	145 (33.0)	141 (32.0)	59 (30.3)	63 (32.3)
Parity				
0	90 (20.5)	102 (23.2)	37 (19.0)	45 (23.1)
1–2	166 (37.7)	143 (32.5)	77 (39.5)	62 (31.8)
3 or more	184 (41.8)	195 (44.3)	81 (41.5)	88 (45.1)
Weight, kg	58.8 ± 9.09	58.6 ± 8.80	58.3 ± 7.39	58.9 ± 9.47
Height, cm	163 ± 5.92	163 ± 5.95	163 ± 5.96	163 ± 5.80
BMI, kg/m^2^	22.2 ± 3.00	22.1 ± 2.95	21.9 ± 2.49	22.2 ± 3.21
MUAC, mm	263 ± 26.9	262 ± 25.4	261 ± 22.3	262 ± 25.9
Subscapular skinfold thickness, mm	12.1 ± 5.83	12.0 ± 5.70	11.7 ± 4.53	12.0 ± 5.63
Tripital skinfold thickness, mm	11.7 ± 4.73	11.9 ± 4.74	11.6 ± 4.27	12.0 ± 5.10
Arm muscle area, cm^2^^,^^5^	40.8 ± 6.75	40.6 ± 6.58	40.2 ± 5.76	40.4 ± 5.75
Arm fat area, cm^2^^,^^5^	14.6 ± 7.21	14.8 ± 6.92	14.1 ± 5.51	14.7 ± 7.17
Arm fat index^5^	25.3 ± 7.22	25.9 ± 7.36	25.4 ± 6.82	25.8 ± 7.51
**Newborn**				
Sex, female	195 (47.1)	207 (51.0)	91 (47.2)	93 (48.2)
Age, days	18.1 ± 3.69	18.0 ± 4.14	18.0 ± 3.83	17.9 ± 3.80

^1^Values are frequencies (percentages) or means ± SDs.

^2^Assessed using FANTA/USAID’s Household Food Insecurity Access Scale.

^3^Protected well, borehole, pipe, or bottled water were considered improved water sources.

^4^Flush toilet connected to local sewage or septic tank, or pit latrine with slab and/or ventilation were considered improved sanitation facilities.

^5^Arm muscle area = (mid-upper arm circumference*π)^2^/(4*π); arm fat area = ((mid-upper arm circumference)^2^/(4* π))—arm muscle area; arm fat index = arm fat area/((mid-upper arm circumference)^2^/(4* π)).

BMI, body mass index; MUAC, mid-upper arm circumference; SD, standard deviation.

Prenatal micronutrient-fortified BEP supplementation resulted in a significantly higher FFMI among mothers (adjusted MD: 0.45; 95% CI: 0.05, 0.84; *P* = 0.026) and newborns (adjusted MD: 0.28; 95% CI: 0.06, 0.50; *P* = 0.012) (**Table 2**). However, we did not find statistically significant group differences in other maternal and newborn body composition variables including FMI, %FFM, and %FM, as well as in anthropometric indices such as maternal BMI and newborn length-for-age, weight-for-age, and weight-for-length *z*-scores. In CACE analysis including only participants with a BEP adherence of at least 75%, we found that prenatal BEP additionally resulted in significantly higher %FFM in mothers (adjusted MD: 1.44; 95% CI: 0.15, 2.73; *P* = 0.029) and newborns (adjusted MD: 2.31; 95% CI: 0.72, 3.89; *P* = 0.004), and lower %FM in mothers (adjusted MD: −1.44; 95% CI: −2.73, −0.15; *P* = 0.029) and newborns (adjusted MD: −2.31; 95% CI: −3.89, −0.72; *P* = 0.004) (**S3 Table**).

In a subgroup analysis, the positive effect of prenatal BEP on maternal FFMI was found among mothers who belonged to food-secure households (adjusted MD: 1.07; 95% CI: 0.29, 1.86; *P* = 0.008; *P*_interaction_ = 0.038) and who had higher BMI (BMI ≥21.0 kg/m^2^) (adjusted MD: 0.88; 95% CI: 0.29, 1.47; *P* = 0.004; *P*_interaction_ = 0.010) and MUAC (MUAC ≥23 cm) (adjusted MD: 1.54; 95% CI: 0.74, 2.34; *P* < 0.001; *P*_interaction_ = 0.004) at study inclusion (**Table 3**). We did not observe effect modification of BEP supplementation by any of the above variables on maternal FMI or newborn FFMI and FMI (**Table 4**).

**Table 2:** Effect of prenatal micronutrient-fortified BEP supplementation on maternal and newborn body composition^1^.

Characteristics	Control	Intervention	Unadjusted difference (95% CI)	*P*	Adjusted difference (95% CI)	*P*
**Maternal (*n*)**	**185**	**186**				
FFMI (kg/m^2^)	17.5 ± 1.78	17.9 ± 2.10	0.50 (0.09, 0.90)	0.015	0.45 (0.05, 0.84)	0.026
FMI (kg/m2)	5.14 ± 2.23	4.99 ± 2.18	−0.15 (−0.59, 0.29)	0.496	−0.22 (−0.58, 0.14)	0.237
%FFM	77.7 ± 7.87	78.6 ± 7.41	0.93 (−0.57, 2.43)	0.223	1.08 (−0.22, 2.39)	0.103
%FM	22.3 ± 7.87	21.4 ± 7.41	−0.93 (−2.43, 0.57)	0.223	−1.08 (−2.39, 0.22)	0.103
BMI (kg/m^2^)	22.6 ± 2.29	22.7 ± 2.42	0.11 (−0.38, 0.61)	0.653	0.09 (−0.32, 0.51)	0.657
**Newborn (*n*)**	**368**	**352**				
FFMI (kg/m^2^)	12.6 ± 1.53	12.9 ± 1.57	0.29 (0.07, 0.51)	0.011	0.28 (0.06, 0.50)	0.012
FMI (kg/m^2^)	0.95 ± 1.33	0.86 ± 1.30	−0.15 (−0.33, 0.04)	0.122	−0.13 (−0.32, 0.05)	0.146
%FFM	93.2 ± 9.41	93.9 ± 9.37	1.05 (−0.27, 2.37)	0.119	0.97 (−0.33, 2.28)	0.143
%FM	6.78 ± 9.41	6.11 ± 9.37	−1.05 (−2.37, 0.27)	0.119	−0.97 (−2.28, 0.33)	0.143
Length-for-age z-score	−0.70 ± 1.11	−0.62 ± 0.99	0.08 (−0.07, 0.23)	0.287	0.07 (−0.07, 0.21)	0.327
Weight-for-age z-score	−0.56 ± 0.95	−0.44 ± 0.90	0.10 (−0.03, 0.24)	0.121	0.11 (−0.02, 0.23)	0.094
Weight-for-length z-score	−0.24 ± 1.10	−0.12 ± 1.03	0.12 (−0.03, 0.27)	0.105	0.13 (−0.02, 0.27)	0.080

^1^Values are means ± SDs. Unadjusted and adjusted group differences were estimated by fitting linear regression models. All models contained the health center and randomization block as covariates to take into account clustering of our data by the study design. Adjusted models contained additional covariates such as maternal age, height, arm fat index, hemoglobin concentration and parity at study enrollment, household size, wealth index and food insecurity status, and number of postpartum days before the body composition measurement.

BEP, balanced energy-protein

BMI, body mass index

CI, confidence interval

FFMI, fat-free mass index

%FFM, fat-free mass as percentage of total body weight

FMI, fat-mass index

%FM, fat-mass as percentage of total body weight

SD, standard deviation.

**Table 3:** Subgroup analyses of the efficacy of prenatal micronutrient-fortified BEP supplementation on maternal and newborn FFMI^1^.

Subgroup factor		Control		Intervention	Unadjusted difference (95% CI)	*P*	Adjusted difference (95% CI)	*P*
	*n*	Mean ± SD	*n*	Mean ± SD				
**Maternal**								
Household food security						0.108		0.038
Food secure	76	17.5 ± 1.65	81	18.3 ± 2.20	0.87 (0.13, 1.62)	0.022	1.07 (0.29, 1.86)	0.008
Food insecure	109	17.5 ± 1.88	105	17.6 ± 1.97	0.40 (−0.16, 0.96)	0.157	0.23 (−0.34, 0.81)	0.427
Maternal BMI						0.014		0.010
≥21.0 kg/m^2^	110	17.9 ± 1.83	116	18.7 ± 2.11	0.93 (0.36, 1.50)	0.002	0.88 (0.29, 1.47)	0.004
<21.0 kg/m^2^	75	16.9 ± 1.53	70	16.6 ± 1.27	−0.29 (−0.82, 0.23)	0.271	−0.47 (−1.03, 0.09)	0.099
Maternal MUAC						0.006		0.004
≥23 cm	82	17.8 ± 1.98	76	18.9 ± 2.25	1.52 (0.77, 2.27)	<0.001	1.54 (0.74, 2.34)	<0.001
<23 cm	103	17.2 ± 1.56	110	17.2 ± 1.67	0.02 (−0.44, 0.49)	0.924	−0.15 (−0.61, 0.31)	0.530
**Newborn**								
Household food security						0.498		0.496
Food secure	150	12.6 ± 1.55	147	12.9 ± 1.68	0.38 (−0.01, 0.78)	0.059	0.31 (−0.10, 0.71)	0.135
Food insecure	221	12.7 ± 1.60	206	12.9 ± 1.64	0.27 (−0.05, 0.59)	0.103	0.27 (−0.06, 0.59)	0.108
Maternal BMI						0.452		0.285
≥21.0 kg/m^2^	230	12.8 ± 1.53	222	13.0 ± 1.64	0.18 (−0.12, 0.48)	0.243	0.14 (−0.17, 0.44)	0.371
<21.0 kg/m^2^	141	12.4 ± 1.62	132	12.7 ± 1.66	0.40 (−0.01, 0.81)	0.057	0.48 (0.07, 0.90)	0.023
Maternal MUAC						0.736		0.892
≥23 cm	170	12.7 ± 1.59	145	13.0 ± 1.75	0.30 (−0.08, 0.68)	0.117	0.24 (−0.14, 0.62)	0.218
<23 cm	201	12.6 ± 1.57	209	12.8 ± 1.58	0.24 (−0.08, 0.55)	0.137	0.25 (−0.07, 0.57)	0.122

^1^Linear regression models were fitted to test the interaction between study arm and a subgroup factor at *P* < 0.10. All models contained the health center and randomization block to account for clustering by the study design. Adjusted models contained additional covariates such as maternal age, height, arm fat index, hemoglobin concentration and parity at study enrollment, household size, wealth index and food security status, and number of postpartum days before the body composition measurement.

BEP, balanced energy-protein

BMI, body mass index

CI, confidence interval

FFMI, fat-free mass index

MUAC, mid-upper arm circumference

SD, standard deviation.

**Table 4:** Subgroup analyses of the efficacy of prenatal micronutrient-fortified BEP supplementation on maternal and newborn FMI^1^.

Subgroup factor		Control		Intervention	Unadjusted difference (95% CI)	*P*	Adjusted difference (95% CI)	*P*
	*n*	Mean ± SD	*n*	Mean ± SD				
**Maternal**								
Household food security						0.720		0.892
Food secure	76	5.39 ± 2.45	***81***	5.13 ± 2.37	−0.29 (−1.21, 0.63)	0.536	−0.12 (−0.87, 0.64)	0.758
Food insecure	109	4.96 ± 2.07	105	4.88 ± 2.03	−0.27 (−0.86, 0.31)	0.354	−0.30 (−0.83, 0.23)	0.272
Maternal BMI						0.277		0.112
≥21.0 kg/m^2^	110	5.91 ± 2.40	116	5.53 ± 2.44	−0.58 (−1.28, 0.13)	0.107	−0.59 (−1.21, 0.03)	0.063
<21.0 kg/m^2^	75	4.00 ± 1.31	70	4.08 ± 1.20	0.14 (−0.34, 0.62)	0.570	0.11 (−0.38, 0.61)	0.652
Maternal MUAC						0.981		0.726
≥23 cm	82	6.11 ± 2.69	76	6.05 ± 2.54	−0.46 (−1.48, 0.56)	0.371	−0.61 (−1.54, 0.32)	0.198
<23 cm	103	4.36 ± 1.39	110	4.25 ± 1.***51***	−0.10 (−0.51, 0.32)	0.649	−0.11 (−0.46, 0.25)	0.552
**Newborn**								
Household food security						0.643		0.783
Food secure	149	0.95 ± 1.31	146	0.77 ± 1.28	−0.19 (−0.51, 0.13)	0.249	−0.12 (−0.44, 0.20)	0.457
Food insecure	221	0.90 ± 1.44	205	0.90 ± 1.38	−0.11 (−0.38, 0.17)	0.438	−0.12 (−0.39, 0.16)	0.406
Maternal BMI						0.581		0.906
≥21.0 kg/m^2^	229	0.95 ± 1.42	221	0.87 ± 1.31	−0.03 (−0.29, 0.22)	0.805	−0.04 (−0.30, 0.21)	0.742
<21.0 kg/m^2^	141	0.88 ± 1.34	131	0.81 ± 1.38	−0.11 (−0.46, 0.22)	0.509	−0.05 (−0.40, 0.29)	0.760
Maternal MUAC						0.951		0.753
≥23 cm	169	1.06 ± 1.40	143	0.99 ± 1.39	−0.09 (−0.42, 0.23)	0.568	−0.09 (−0.42, 0.24)	0.590
<23 cm	201	0.81 ± 1.37	209	0.75 ± 1.29	−0.08 (−0.34, 0.19)	0.572	−0.05 (−0.31, 0.22)	0.730

^1^Linear regression models were fitted to test interaction between study arm and a subgroup factor at *P* < 0.10. All models contained the health center and randomization block to account for clustering by the study design. Adjusted models contained additional covariates such as maternal age, height, arm fat index, hemoglobin concentration and parity at study enrollment, household size, wealth index and food security status, and number of postpartum days before the body composition measurement.

BEP, balanced energy-protein

BMI, body mass index

CI, confidence interval

FMI, fat-mass index

MUAC, mid-upper arm circumference

SD, standard deviation.

When exploring the treatment effects on maternal FFMI by maternal BMI percentile at study inclusion, we observed a larger BEP treatment effect at higher percentiles of the maternal BMI distribution (**S1 Fig**). A more complex pattern emerged for newborn FFMI with stronger BEP effects at low and high BMI percentiles. The treatment effects of BEP on maternal and newborn FMI across maternal BMI percentiles showed a less consistent pattern (**S2 Fig**). The relationship between maternal and newborn FFMI also showed no evidence of a tradeoff between newborn and mother of the BEP treatment effect on FFMI (**S3 Fig**). Lastly, we found a positive relationship between maternal and newborn FFMI and FMI and birth anthropometry, such as birth weight, MUAC, and ponderal index (**S4 Table**).

## Discussion

Our study found that prenatal BEP led to a higher maternal and newborn FFMI without significant effects on FMI. The positive effect of prenatal BEP on maternal FFMI was found to be larger among mothers with a better nutritional status at baseline (i.e., BMI ≥21.0 kg/m^2^ or MUAC ≥23 cm).

Our results are in contrast to those from the MISAME-II trial conducted in the same health district between 2006 and 2008. MISAME-II found elevated cord blood leptin concentration, indicating larger newborn FM accretion [13,14], in the group who received prenatal MMN-fortified LNS supplementation as compared to MMN alone [12]. Conversely, our findings suggest that instead of pathways favoring FM accretion, the small positive effects of BEP observed on newborn size at birth [9] are driven by other physiologic and metabolic mechanisms. These may include increased nutrient availability to the placenta [30,31], improvements in maternal hematologic profile [32], favorable maternal immune responses and related lower burden of infectious diseases, asymptomatic inflammation and oxidative stress [31,33,34], or other epigenetic gene regulation pathways [31,35,36]. A study in a cohort of Kenyan women and their newborns also found positive associations between newborn birth weight and maternal TBW and FFM, but not with FM, assessed using isotope dilution technique [37]. On the other hand, a cohort study in pregnant women from an urban slum in India reported that maternal FFM in the third trimester was negatively associated with LBW, while FM was negatively associated with the occurrence of SGA [38].

A number of trials have evaluated the effects of prenatal LNS on maternal and newborn outcomes [6,39–42], but data on newborn body composition are lacking. A prenatal LNS supplementation made from peanut butter (400 kcal/day) provided to a small sample of primigravid women in Pakistan did not affect postpartum maternal body composition, using bioelectrical impedance scale, as compared to the control supplement made from wheat flakes and skimmed milk (138 kcal/day) [43]. Another trial evaluated the effects of an energy-protein food supplement provided starting either from early (approximately 9 weeks of gestation) or later pregnancy (approximately 20 weeks of gestation), in combination with IFA or MMN supplements, in a large-sample of women in Bangladesh. The study found that offspring body composition using a bioelectrical impedance scale at the age 54 months was not affected by the prenatal supplementation [44]. Similarly, LNS supplementation provided to mothers during pregnancy and lactation and continued in their children aged 6 to 18 months did not result in any long-term effect on child FFM and FM at the age of 4 to 6 years as compared to control supplements containing IFA and calcium or MMN [45].

Various possible explanations can be forwarded for our finding that prenatal BEP in mothers and their newborns led to FFM accretion rather than FM accretion. First, the composition of the BEP supplement used could possibly influence the balance between FFM and FM tissue accretions. Diets and supplements containing animal-source proteins, especially those from dairy products, support lean mass accretion better than diets or supplements containing only plant-based protein sources [46]. Unlike the LNS supplement used in MISAME-II, which contained only plant-based protein sources (soy and peanut protein) with a protein digestibility corrected amino acid score of 0.9, the BEP in the current study contained both plant- and animal-source protein (i.e., soy: 61%, peanut: 15%, and milk: 25%) with an amino acid score of 1.1. Furthermore, the BEP provided a range of micronutrients that are essential for the synthesis of maternal and fetal tissues [47], which can be reflected in improved FFM accretion. Contrary to the current study where control mothers were given IFA, MISAME-II provided MMN to the control group which may have supported FFM accretion and thereby reduced any relative impact of the LNS on FFM. Second, the effects of prenatal BEP on body composition could depend on maternal baseline nutritional status and energy balance during pregnancy. Extra energy intake is required during the second and third trimester of gestation to support adequate gestational weight gain and the increase in basal metabolic rate [48]. In the absence of an adequate energy balance, FFM preservation in essential maternal and fetal tissue compartments could be prioritized over FM preservation. Given the lack of impact of BEP on maternal fat mass in mothers with higher BMI at baseline, we hypothesize that the additional energy provided by the BEP may have been insufficient to fill the additional energy requirement due to pregnancy to result in higher postpartum fat mass. A 24 h recall survey in a subsample of the study participants found that the median energy intake, including BEP, was 2,329 kcal/day during the lean season [18], which is lower than our estimated energy requirement of mothers. The mean daily energy requirement based on maternal weight and a physical activity level of 1.79 (estimated in MISAME-II mothers during the lean season [49]) was estimated at 2,430 kcal without including the additional daily requirements of 290 kcal and 465 kcal during the second and third trimester of pregnancy [50]. A previous dietary recall survey in the same health district also revealed that pregnant women did not have a higher energy intake than non-pregnant women, which suggests that the additional energy requirement due to pregnancy was not accounted for by their dietary intake [17].

Subgroup analyses showed that prenatal BEP was more efficacious in increasing maternal FFM index in the women from food secure households or with a more adequate energy balance (i.e., baseline BMI ≥21.0 kg/m^2^ or MUAC ≥23 cm). These findings are in line with our previous finding that BEP was more efficacious in reducing SGA prevalence among mothers with a more adequate baseline nutritional status (e.g., nonanemic, higher MUAC) [9]. One possible explanation for the lack of significant effect on FFM among women with low BMI could be due to a limited amino acids supply from endogenous breakdown to meet the demands of pregnancy. A study in a small sample of low BMI Indian women showed that BEP supplementation using 300 kcal/d and 15 g protein/d starting from gestational age of 12 weeks did not result in improved whole-body protein synthesis, improved fluxes in the methyl group precursors serine and glycine, or better pregnancy outcomes including higher gestational weight gain and newborn weight [51]. These findings might suggest extending BEP supplementation to the pre-conception period to leverage the efficacy of prenatal BEP on optimal maternal and newborn outcomes.

The observed effects of prenatal BEP on newborn FFMI can be seen in relation to the subsequent modest effect of prenatal BEP on linear growth achieved at 6 months of age (0.11 z-score; 95% CI: 0.01, 0.21; *p* = 0.032) that we reported previously [52]. In Ethiopia, Admassu and colleagues [53] found that newborn FFM, but not FM, was positively associated with height-for-age z-score at 2 years of age. Furthermore, FFM in the first month of life was associated with linear growth at the age of 1 year [54]. A Danish cohort also found that newborns with more FFM were on average both taller and heavier at 3 years of age [55].

A major strength of our study was the direct measurement of body composition using the deuterium dilution method, which enables robust assessment that can be implemented at scale in a rural field setting. We were also able to achieve a high adherence to both the BEP and IFA supplements through daily home visits by community-based study workers who observed supplement intake. A dietary intake survey conducted in a subsample of the study participants also ruled out any substitution effect of the BEP on usual dietary patterns [18]. The CACE analysis evaluating the intervention’s effects among subjects with better compliance also confirmed our findings in the main analysis.

The study has several limitations that need to be addressed. About 18% of the newborn outcome data was missing mainly due to saliva samples not being collected or unreadable identification numbers on cryotubes, whereas we anticipated a 10% loss when designing the study. However, the occurrence of missing data was not specific to any of the study group and maternal and household characteristics were comparable between study groups. Therefore, we argue that the missing data are unlikely to have biased group comparisons. Maternal body composition using the deuterium dilution method was not measured at baseline. As such, we were only able to consider baseline adjustment in our models for proxy variables of body composition such as BMI and arm fat index. Lastly, having follow-up body composition measurements would have revealed whether observed effects are sustained in the long term.

In conclusion, our study demonstrated that prenatal BEP supplementation has positive effects on newborn and maternal FFM accretion without significant alteration of FMI. The absence of any effects of BEP on fat mass accretion in both newborn and mother addresses the possible concern that prenatal BEP would lead to excess adiposity and associated long-term health risks.

## Supporting information

S1 Table Nutritional values of the ready-to-use supplementary food for pregnant women. (DOCX)

S2 Table Comparison of participants between the MISAME-III study and the body composition sub-studies. (DOCX)

S3 Table Effect of prenatal micronutrient-fortified BEP supplementation on maternal and newborn body composition using CACE estimates. (DOCX)

S4 Table Relationship between newborn and maternal FFMI and FMI and birth anthropometry. (DOCX)

S1 Fig Treatment effect on maternal and newborn FFMI across the distribution of maternal BMI at baseline. The estimated difference in FFMI between the intervention and control groups is plotted as a function of the percentiles of maternal BMI. The zero line indicates no efficacy of BEP. The positive y values indicate a higher FFMI in the intervention group, and the negative y values indicate a lower FFMI, with upper and lower 95% confidence bands. BMI, body mass index; FFMI, fat-free mass index.
(TIF)

S2 Fig Treatment effect on maternal and newborn FMI across the distribution of maternal BMI at baseline. The estimated difference in FMI between the intervention and control groups is plotted as a function of the percentiles of maternal BMI. The zero line indicates no efficacy of BEP. The positive y values indicate a higher FMI in the intervention group, and the negative y values indicate a lower FMI, with upper and lower 95% confidence bands. BMI, body mass index; FFI, fat-mass index.
(TIF)

S3 Fig Locally weighted scatterplot smoothing plotting the relationship between newborn and maternal FFMI. Linear regression model was fitted to estimate the beta and 95% CIs for the relationship between newborn and maternal FFMI.
(TIF)

S1 CONSORT Checklist CONSORT checklist of the manuscript. (PDF)

S1 Statistical Analysis Plan Impact of a prenatal and postnatal balanced energy protein supplement on birth size and postnatal child growth in Burkina Faso. (PDF)

## Abbreviations

BEP: balanced energy-protein
BMI: body mass index
CI: confidence interval
CACE: complier average causal effect
D2O: deuterium oxide
EAR: estimated average requirement
FFMI: fat-free mass index
%FFM: fat-free mass as percentage of total body weight
FMI: fat-mass index
%FM: fat-mass as percentage of total body weight
FTIR: Fourier-transformed infrared
IFA: iron-folic acid
LBW: low birth weight
LNS: lipid-based nutrient supplement
MD: mean difference
MISAME: MIcronutriments pour la SAnté de la Mère et de l’Enfant
MMN: multimicronutrient tablet
MUAC: mid-upper arm circumference
RR: relative risk
SD: standard deviation
SGA: small-for-gestational age, TBW, total body water
