# Supplementary material for: Effect of prenatal micronutrient-fortified balanced energy-protein supplementation on maternal and newborn body composition: A sub-study from the MISAME-III randomized controlled efficacy trial in rural Burkina Faso
Source: PLoS Med. 2023 Jul 24;20(7):e1004242. doi: 10.1371/journal.pmed.1004242 (PMC10406330; doi:10.1371/journal.pmed.1004242)
Supplement: S1 Table — (DOCX) [file pmed.1004242.s001.docx]

**Table S1. Nutritional values of the ready-to-use supplementary food for pregnant women^1^**

|  | **Mean for 72g (serving size)** |
| --- | --- |
| Total energy (kcal) | 393 |
| Lipids (g) | 26 |
| Linoleic acid (g) | 3.9 |
| α-Linoleic acid (g) | 1.3 |
| Proteins (g) | 14.5 |
| Carbohydrates (g) | 23.3 |
| Calcium (mg) | 500 |
| Copper (mg) | 1.3 |
| Phosphorus (mg) | 418 |
| Iodine (µg) | 250 |
| Iron (mg) | 22 |
| Selenium (µg) | 65 |
| Manganese (mg) | 2.1 |
| Magnesium (mg) | 73 |
| Potassium (mg) | 562 |
| Zinc (mg) | 15 |
| Vitamin A (µg RE)^2^ | 770 |
| Thiamin (mg) | 1.4 |
| Riboflavin (mg) | 1.4 |
| Niacin (mg) | 15 |
| Vitamin B5 (mg) | 7 |
| Vitamin B6 (mg) | 1.9 |
| Folic acid (µg) | 400 |
| Vitamin B12 (mg) | 2.6 |
| Vitamin C (mg) | 100 |
| Vitamin D (µg cholecalciferol)^3^ | 15 |
| Vitamin E (mg α-tocopherol)^4^ | 18 |
| Vitamin K (µg) | 72 |

^1^Ingredients: vegetable oils (rapeseed, palm, soy in varying proportions), defatted soy flour, skimmed milk powder, peanuts, sugar, maltodextrin, soy protein isolate, vitamin and mineral complex, stabilizer (fully hydrogenated vegetable fat, mono and diglycerides).

^2^1 µg vitamin A RE = 3.333 IU vitamin A.

^3^1 μg cholecalciferol = 40 IU vitamin D.

^4^1 mg α-tocopherol = 2,22 IU vitamin E.

IU, international unit; RE, retinol equivalent.
