# Supplementary material for: Effect of prenatal micronutrient-fortified balanced energy-protein supplementation on maternal and newborn body composition: A sub-study from the MISAME-III randomized controlled efficacy trial in rural Burkina Faso
Source: PLoS Med. 2023 Jul 24;20(7):e1004242. doi: 10.1371/journal.pmed.1004242 (PMC10406330; doi:10.1371/journal.pmed.1004242)
Supplement: S2 Table — (DOCX) [file pmed.1004242.s002.docx]

**Table S2. Comparison of participants between the MISAME-III study and the body composition sub-study^1^**

| **Characteristics** | **MISAME study (*n* = 1788)** | **Infant subsample (*n* = 880)** | **Maternal subsample (*n* = 390)** |
| --- | --- | --- | --- |
| **Health center catchment area** |  |  |  |
| Boni | 392 (21.9) | 217 (24.7) | 95 (24.4) |
| Dohoun | 192 (10.7) | 86 (9.80) | 48 (12.3) |
| Dougoumato II | 326 (18.2) | 167 (19.0) | 66 (16.9) |
| Karaba | 187 (10.5) | 87 (9.90) | 38 (9.70) |
| Kari | 331 (18.5) | 168 (19.1) | 69 (17.7) |
| Koumbia | 360 (20.1) | 155 (17.6) | 74 (19.0) |
| **Household level** |  |  |  |
| Wealth index, 0 to 10 points | 4.59 ± 1.75 | 4.60 ± 1.76 | 4.58 ± 1.72 |
| Household food insecurity^2^ | 978 (54.7) | 516 (58.6) | 229 (56.2) |
| Improved primary water source^3^ | 1116 (62.4) | 544 (61.8) | 229 (58.7) |
| Improved sanitation facility^4^ | 1072 (60.0) | 508 (57.7) | 219 (56.2) |
| Household size | 6.19 ± 4.33 | 6.37 ± 4.53 | 6.46 ± 4.52 |
| Polygamous households | 576 (32.2) | 258 (29.3) | 119 (30.5) |
| **Maternal** |  |  |  |
| Age, years | 25.0 ± 6.19 | 24.8 ± 6.13 | 24.8 ± 6.04 |
| Ethnic group |  |  |  |
| Bwaba | 1027 (57.4) | 518 (58.9) | 220 (56.4) |
| Mossi | 624 (34.9) | 291 (33.1) | 143 (36.7) |
| Others | 137 (7.66) | 71 (8.07) | 27 (6.92) |
| Religion pregnant woman |  |  |  |
| Muslim | 755 (42.2) | 362 (41.1) | 171 (43.8) |
| Animist | 413 (23.1) | 197 (22.4) | 80 (20.5) |
| Protestant | 309 (17.3) | 174 (19.8) | 77 (19.7) |
| Catholic | 246 (13.8) | 116 (13.2) | 55 (14.1) |
| No religion, no animist | 63 (3.50) | 30 (3.40) | 7 (1.8) |
| Primary education and above | 749 (41.9) | 371 (42.2) | 178 (45.6) |
| Gestational age, weeks | 11.5 ± 4.06 | 10.9 ± 3.82 | 10.9 ± 3.91 |
| Trimester of gestation |  |  |  |
| First | 1119 (62.6) | 598 (68.0) | 263 (67.4) |
| Second | 669 (37.4) | 282 (32.0) | 127 (32.6) |
| Parity |  |  |  |
| 0 | 401 (22.4) | 206 (23.4) | 86 (22.1) |
| 1-2 | 620 (34.7) | 307 (34.9) | 139 (35.6) |
| 3 or more | 767 (42.9) | 367 (41.7) | 165 (42.3) |
| Weight, kg | 58.1 ± 8.67 | 58.6 ± 8.95 | 58.4 ± 8.25 |
| Height, cm^5^ | 163 ± 5.98 | 163 ± 5.90 | 163 ± 5.78 |
| BMI, kg/m^2^ | 22.0 ± 2.87 | 22.1 ± 3.02 | 22.0 ± 2.78 |
| Mid-upper arm circumference, mm | 262 ± 26.6 | 262 ± 26.4 | 261 ± 23.8 |
| Subscapular skinfold thickness, mm | 12.0 ± 5.52 | 12.07 ± 5.81 | 11.7 ± 4.81 |
| Tripital skinfold thickness, mm | 11.9 ± 4.81 | 11.9 ± 4.79 | 11.6 ± 4.40 |
| Arm muscle area, cm^2,5^ | 40.5 ± 6.68 | 40.7 ± 6.62 | 40.3 ± 5.75 |
| Arm fat area, cm^2,5^ | 14.8 ± 7.21 | 14.6 ± 6.99 | 14.4 ± 6.41 |
| Arm fat index^2^ | 25.8 ± 7.40 | 25.5 ± 7.26 | 25.6 ± 7.18 |
| **Newborn** |  |  |  |
| Sex, female | 832 (49.4) | 406 (49.6) | 181 (47.3) |

^1^Values are frequencies (percentages) or means ± SDs

^2^Assessed using FANTA/USAID’s Household Food Insecurity Access Scale.

^3^Protected well, borehole, pipe or bottled water were considered improved water sources.

^4^Flush toilet connected to local sewage or septic tank, or pit latrine with slab and/or ventilation were considered improved sanitation facilities.

^5^Arm muscle area= (mid-upper arm circumference*π)^2^/(4*π); arm fat area= ((mid-upper arm circumference)^2^/(4* π))- arm muscle area; arm fat index = arm fat area/(( mid-upper arm circumference )^2^/(4* π))
