# Supplementary material for: Effect of prenatal micronutrient-fortified balanced energy-protein supplementation on maternal and newborn body composition: A sub-study from the MISAME-III randomized controlled efficacy trial in rural Burkina Faso
Source: PLoS Med. 2023 Jul 24;20(7):e1004242. doi: 10.1371/journal.pmed.1004242 (PMC10406330; doi:10.1371/journal.pmed.1004242)
Supplement: S3 Table — (DOCX) [file pmed.1004242.s003.docx]

**Table S3. Effect of prenatal micronutrient-fortified BEP supplementation on maternal and newborn body composition using CACE estimates^1^**

| **Characteristics** | **Control** | **Intervention** | **Adjusted difference (95% CI)** | ***P*** |
| --- | --- | --- | --- | --- |
| **Maternal (*n*)** | **185** | **186** |  |  |
| FFMI (kg/m^2^) | 17.5 ± 1.78 | 17.9 ± 2.10 | 0.36 (0.00, 0.71) | 0.051 |
| FMI (kg/m2) | 5.14 ± 2.23 | 4.99 ± 2.18 | -0.22 (-0.52, 0.08) | 0.149 |
| %FFM | 77.7 ± 7.87 | 78.6 ± 7.41 | 1.44 (0.15, 2.73) | 0.029 |
| %FM | 22.3 ± 7.87 | 21.4 ± 7.41 | -1.44 (-2.73, -0.15) | 0.029 |
| BMI (kg/m^2^) | 22.6 ± 2.29 | 22.7 ± 2.42 | 0.31 (-0.09, 0.71) | 0.131 |
| **Newborn (*n*)** | **368** | **352** |  |  |
| FFMI (kg/m^2^) | 12.6 ± 1.53 | 12.9 ± 1.57 | 0.52 (0.23, 0.81) | <0.001 |
| FMI (kg/m^2^) | 0.95 ± 1.33 | 0.86 ± 1.30 | -0.15 (-0.40, 0.09) | 0.218 |
| %FFM | 93.2 ± 9.41 | 93.9 ± 9.37 | 2.31 (0.72, 3.89) | 0.004 |
| %FM | 6.78 ± 9.41 | 6.11 ± 9.37 | -2.31 (-3.89, -0.72) | 0.004 |
| Length-for-age z-score | -0.70 ± 1.11 | -0.62 ± 0.99 | 0.03 (-0.13, 0.20) | 0.694 |
| Weight-for-age z-score | -0.56 ± 0.95 | -0.44 ± 0.90 | 0.13 (-0.01, 0.28) | 0.077 |
| Weight-for-length z-score | -0.24 ± 1.10 | -0.12 ± 1.03 | 0.13 (-0.05, 0.31) | 0.151 |

^1^Values are means ± SDs. The CACE estimates compared subjects with at least 75% of BEP adherence and ‘inferred compliers’ (would be compliers) in the control groups. We applied a latent class regression modelling to simultaneously fit models for compliance and for the intervention effect. Compliance status was predicted by covariates such as household food insecurity, access to improved water and sanitation, number of under-five children and number of job activities by the women and the household head, while the effects of BEP on the study outcomes were estimated by adjusting for maternal age, height, arm fat index, hemoglobin concentration and parity at study enrollment, household size, asset index and food insecurity, and number of postpartum days before the body composition measurement. BEP, balanced energy-protein; BMI, body mass index; CACE, complier average causal effect; FFMI, fat-free mass index, %FFM, fat-free mass as percentage of total body weight; FMI, fat-mass index; %FM, fat-mass as percentage of total body weight.
