# Supplementary material for: Effect of prenatal micronutrient-fortified balanced energy-protein supplementation on maternal and newborn body composition: A sub-study from the MISAME-III randomized controlled efficacy trial in rural Burkina Faso
Source: PLoS Med. 2023 Jul 24;20(7):e1004242. doi: 10.1371/journal.pmed.1004242 (PMC10406330; doi:10.1371/journal.pmed.1004242)
Supplement: S4 Table — (DOCX) [file pmed.1004242.s004.docx]

**Table S4. Relationship between newborn and maternal FFMI and FMI and birth anthropometry^1^**

| **Characteristics** | **Birth weight (kg)** | ***P*** | **MUAC (mm)** | ***P*** | **Ponderal index (g/cm^3)^** | ***P*** |
| --- | --- | --- | --- | --- | --- | --- |
| Maternal FFMI (kg/m^2^) | 0.04 (0.02, 0.06) | <0.001 | 0.83 (0.42, 1.25) | <0.001 | 0.17 (0.03, 0.30) | 0.013 |
| Maternal FMI (kg/m^2^) | 0.02 (0.00, 0.03) | 0.071 | 0.49 (0.12, 0.86) | 0.010 | 0.10 (0.02, 0.22) | 0.099 |
| Newborn FFMI (kg/m^2^) | 0.06 (0.04, 0.08) | <0.001 | 0.93 (0.59, 1.27) | <0.001 | 0.35 (0.24, 0.46) | <0.001 |
| Newborn FMI (kg/m2) | 0.05 (0.03, 0.07) | <0.001 | 0.92 (0.51, 1.32) | <0.001 | 0.22 (0.09, 0.36) | 0.001 |

^1^Values are betas (95% CIs) estimating the association between indices using linear regression models. FFMI, fat-free mass index, %FFM, fat-free mass as percentage of total body weight; FMI, fat-mass index; %FM, fat-mass as percentage of total body weight, MUAC, mid upper arm circumference.
