# Supplementary figures and images for: Effect of prenatal micronutrient-fortified balanced energy-protein supplementation on maternal and newborn body composition: A sub-study from the MISAME-III randomized controlled efficacy trial in rural Burkina Faso
Source: PLoS Med. 2023 Jul 24;20(7):e1004242. doi: 10.1371/journal.pmed.1004242 (PMC10406330; doi:10.1371/journal.pmed.1004242)

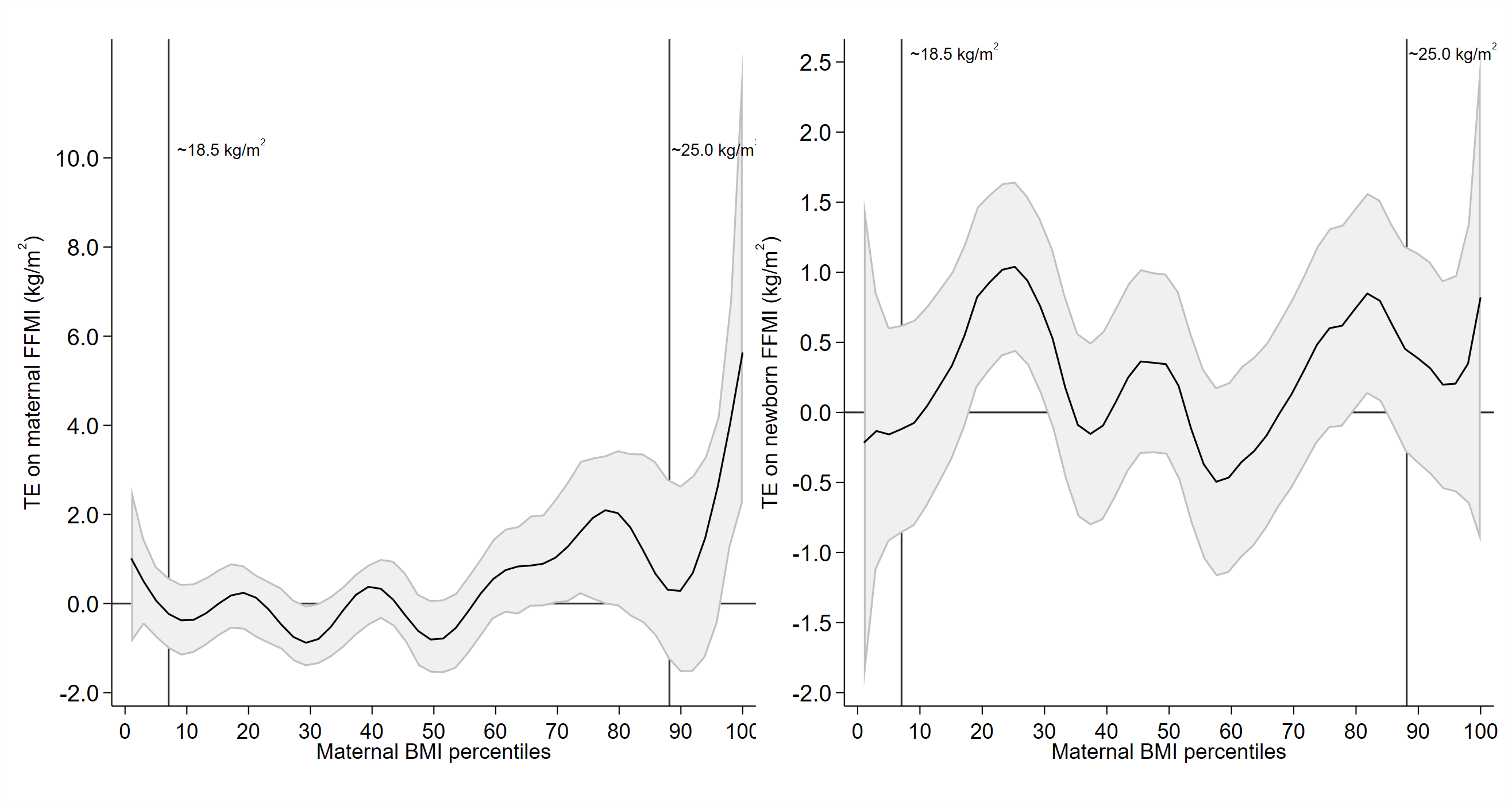

Supplement: S1 Fig — The estimated difference in FFMI between the intervention and control groups is plotted as a function of the percentiles of maternal BMI. The zero line indicates no efficacy of BEP. The positive y values indicate a higher FFMI in the intervention group, and the negative y values indicate a lower FFMI, with upper and lower 95% confidence bands. BMI, body mass index; FFMI, fat-free mass index. (TIF) [file pmed.1004242.s005.tif]

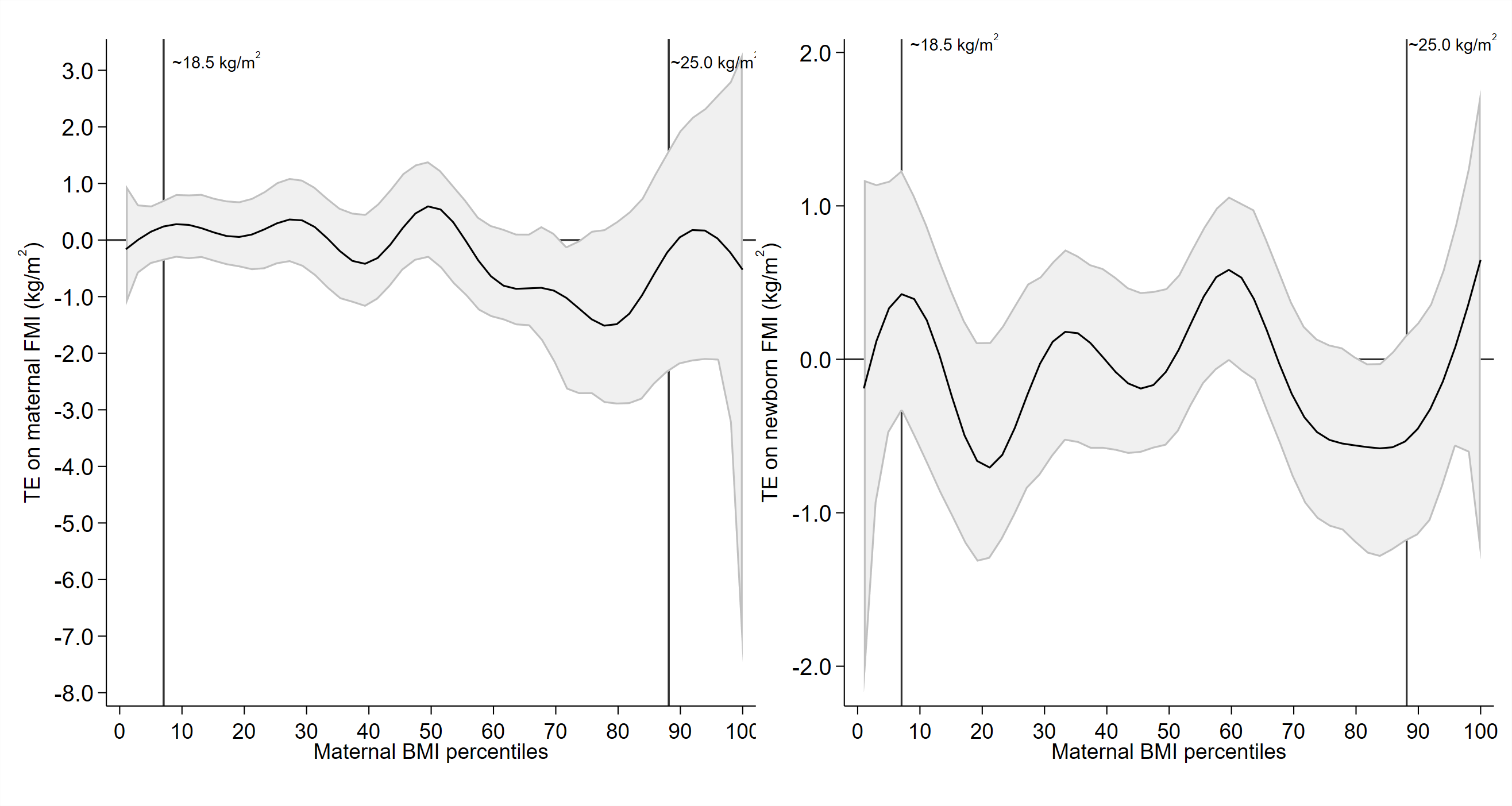

Supplement: S2 Fig — The estimated difference in FMI between the intervention and control groups is plotted as a function of the percentiles of maternal BMI. The zero line indicates no efficacy of BEP. The positive y values indicate a higher FMI in the intervention group, and the negative y values indicate a lower FMI, with upper and lower 95% confidence bands. BMI, body mass index; FFI, fat-mass index. (TIF) [file pmed.1004242.s006.tif]

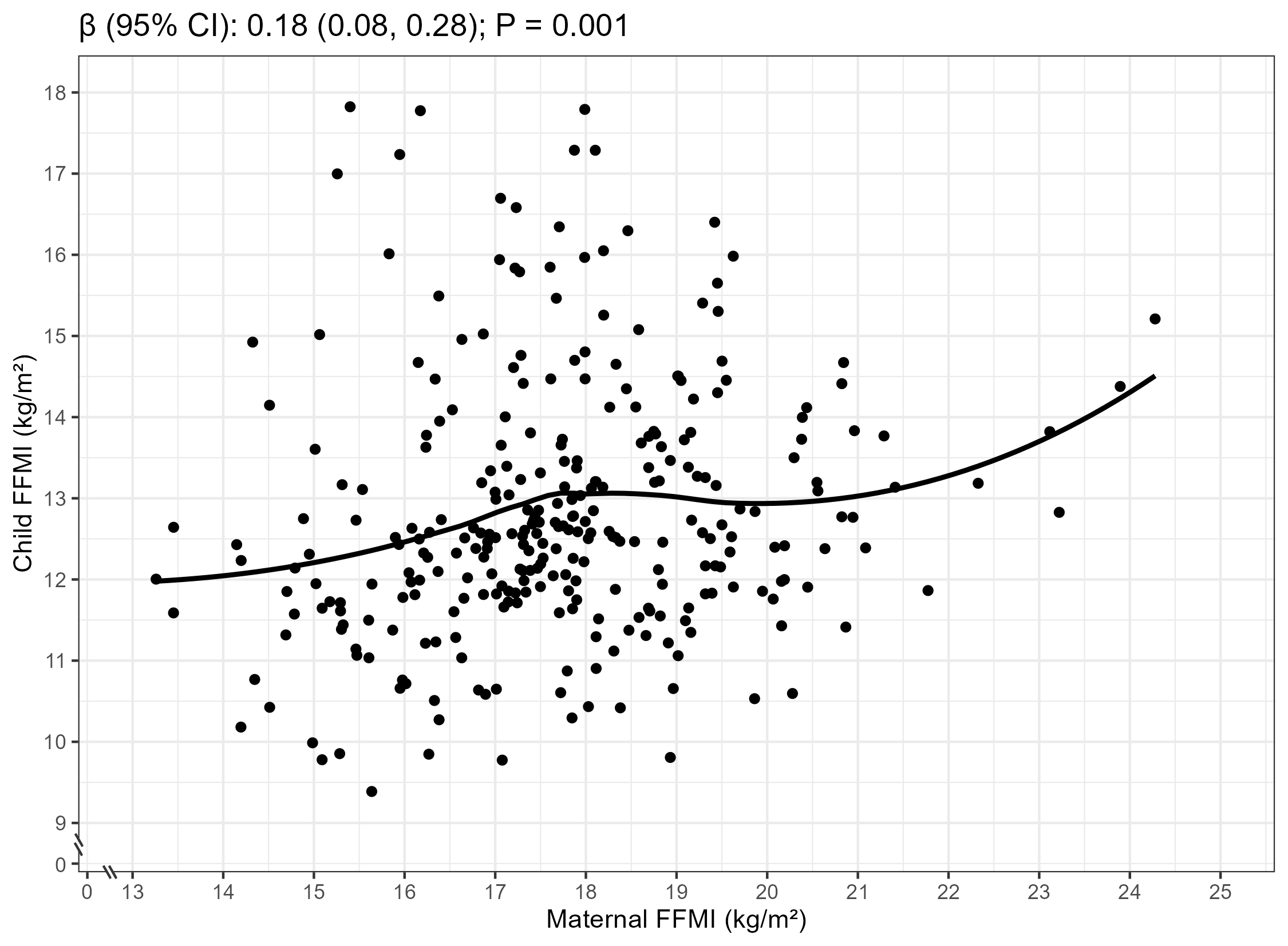

Supplement: S3 Fig — Linear regression model was fitted to estimate the beta and 95% CIs for the relationship between newborn and maternal FFMI. (TIF) [file pmed.1004242.s007.tif]
