# Supplementary material for: Effect of prenatal micronutrient-fortified balanced energy-protein supplementation on maternal and newborn body composition: A sub-study from the MISAME-III randomized controlled efficacy trial in rural Burkina Faso
Source: PLoS Med. 2023 Jul 24;20(7):e1004242. doi: 10.1371/journal.pmed.1004242 (PMC10406330; doi:10.1371/journal.pmed.1004242)
Supplement: S1 CONSORT Checklist — (PDF) [file pmed.1004242.s008.pdf]

CONSORT checklist of the manuscript

Section/Topic	Item No	Checklist item	Reported on page No
**Title and abstract**			
	1a	Identification as a randomised trial in the title	yes
	1b	Structured summary of trial design, methods, results, and conclusions (for specific guidance see CONSORT for abstracts)	Par. 2, 3 & 4
**Introduction**			
Background and objectives	2a	Scientific background and explanation of rationale	Par 1- 3
	2b	Specific objectives or hypotheses	Par 3
**Methods**			
Trial design	3a	Description of trial design (such as parallel, factorial) including allocation ratio	Par. 1 & 2
	3b	Important changes to methods after trial commencement (such as eligibility criteria), with reasons	NA
Participants	4a	Eligibility criteria for participants	Par. 4
	4b	Settings and locations where the data were collected	Par. 2
Interventions	5	The interventions for each group with sufficient details to allow replication, including how and when they were actually administered	Par 1, 3
Outcomes	6a	Completely defined pre-specified primary and secondary outcome measures, including how and when they were assessed	Par 6-9
	6b	Any changes to trial outcomes after the trial commenced, with reasons	NA
Sample size	7a	How sample size was determined	Par 9
	7b	When applicable, explanation of any interim analyses and stopping guidelines	NA
Randomisation:			
Sequence generation	8a	Method used to generate the random allocation sequence	Par 2 & 3
	8b	Type of randomisation; details of any restriction (such as blocking and block size)	Par 2 & 3 (citation for details)
Allocation concealment mechanism	9	Mechanism used to implement the random allocation sequence (such as sequentially numbered containers), describing any steps taken to conceal the sequence until interventions were assigned	Par 2 & 3 (citation for details)
Implementation	10	Who generated the random allocation sequence, who enrolled participants, and who assigned participants to	Par 2 & 3

		interventions	(citation for details) Par 4
Blinding	11a	If done, who was blinded after assignment to interventions (for example, participants, care providers, those assessing outcomes) and how	Par 2 (citation for details)
	11b	If relevant, description of the similarity of interventions	NA
Statistical methods	12a	Statistical methods used to compare groups for primary and secondary outcomes	Par 11 & 12
	12b	Methods for additional analyses, such as subgroup analyses and adjusted analyses	Par 11 & 12
**Results**			
Participant flow (a diagram is strongly recommended)	13a	For each group, the numbers of participants who were randomly assigned, received intended treatment, and were analysed for the primary outcome	Fig 1 & Par 1
	13b	For each group, losses and exclusions after randomisation, together with reasons	Fig 1 & Par 1
Recruitment	14a	Dates defining the periods of recruitment and follow-up	In the methods: Par 4
	14b	Why the trial ended or was stopped	NA
Baseline data	15	A table showing baseline demographic and clinical characteristics for each group	Table 1 & S1
Numbers analysed	16	For each group, number of participants (denominator) included in each analysis and whether the analysis was by original assigned groups	Fig 1, par 1
Outcomes and estimation	17a	For each primary and secondary outcome, results for each group, and the estimated effect size and its precision (such as 95% confidence interval)	Par 2
	17b	For binary outcomes, presentation of both absolute and relative effect sizes is recommended	NA
Ancillary analyses	18	Results of any other analyses performed, including subgroup analyses and adjusted analyses, distinguishing pre-specified from exploratory	Par 3 & 4
Harms	19	All important harms or unintended effects in each group (for specific guidance see CONSORT for harms)	NA
**Discussion**			
Limitations	20	Trial limitations, addressing sources of potential bias, imprecision, and, if relevant, multiplicity of analyses	Par 7
Generalisability	21	Generalisability (external validity, applicability) of the trial findings	Par 2-5
Interpretation	22	Interpretation consistent with results, balancing benefits and harms, and considering other relevant evidence	Par 2-5
**Other information**			
Registration	23	Registration number and name of trial registry	Methods: Par 3
Protocol	24	Where the full trial protocol can be accessed, if available	Methods: Par 2
Funding	25	Sources of funding and other support (such as supply of drugs), role of funders	Cover page
